# Unexplained False Negative Results in Noninvasive Prenatal Testing: Two Cases Involving Trisomies 13 and 18

**DOI:** 10.1155/2015/926545

**Published:** 2015-06-07

**Authors:** R. Hochstenbach, G. C. M. L. Page-Christiaens, A. C. C. van Oppen, K. D. Lichtenbelt, J. J. T. van Harssel, T. Brouwer, G. T. R. Manten, P. van Zon, M. Elferink, K. Kusters, O. Akkermans, J. K. Ploos van Amstel, G. H. Schuring-Blom

**Affiliations:** ^1^Department of Medical Genetics, Division of Biomedical Genetics, University Medical Centre Utrecht, P.O. Box 85090, Mail Stop KC04.084.2, 3508 AB Utrecht, Netherlands; ^2^Department of Obstetrics and Gynecology, University Medical Centre Utrecht, P.O. Box 85090, Mail Stop KE04.123.1, 3508 AB Utrecht, Netherlands

## Abstract

Noninvasive prenatal testing (NIPT) validation studies show high sensitivity and specificity for detection of trisomies 13, 18, and 21. False negative cases have rarely been reported. We describe a false negative case of trisomy 13 and another of trisomy 18 in which NIPT was commercially marketed directly to the clinician. Both cases came to our attention because a fetal anatomy scan at 20 weeks of gestation revealed multiple anomalies. Karyotyping of cultured amniocytes showed nonmosaic trisomies 13 and 18, respectively. Cytogenetic investigation of cytotrophoblast cells from multiple placental biopsies showed a low proportion of nontrisomic cells in each case, but this was considered too small for explaining the false negative NIPT result. The discordant results also could not be explained by early gestational age, elevated maternal weight, a vanishing twin, or suboptimal storage or transport of samples. The root cause of the discrepancies could, therefore, not be identified. The couples involved experienced difficulties in accepting the unexpected and late-adverse outcome of their pregnancy. We recommend that all parties involved in caring for couples who choose NIPT should collaborate to clarify false negative results in order to unravel possible biological causes and to improve the process of patient care from initial counseling to communication of the result.

## 1. Introduction

Noninvasive prenatal testing (NIPT) based on massively parallel sequencing (MPS) of cell-free fetal DNA (cffDNA) fragments in the maternal circulation is rapidly becoming common clinical practice [1–5]. These DNA fragments are derived from apoptotic placental cytotrophoblast cells [4–6]. At approximately 10 weeks of gestation, the fraction of cffDNA fragments in the maternal circulation is about 10–20% of the total cell-free DNA while the remainder is of maternal origin [3–5].

NIPT enables testing for trisomies 13, 18, and 21 in pregnancies that are at elevated risk for aneuploidy, for example, because of maternal age, first trimester combined screening result, or ultrasound abnormalities [3–6]. Prospective studies show that trisomies 18 and 21 can also reliably be detected by NIPT in an unselected obstetric population [7–10]. In both groups the sensitivity and specificity of NIPT are 99-100% for trisomy 21. For trisomy 18, sensitivity is 90–100% and specificity is 99-100%; for trisomy 13, they are 93–100% and 99-100%, respectively [6–10]. Nevertheless, NIPT is not considered a diagnostic test and, in case of a positive NIPT result, follow-up invasive testing by chorionic villi sampling (CVS) or amniocentesis must be offered before a definitive diagnosis can be made [11, 12]. The major reason is that placental DNA is not representative of the fetus in all cases, a phenomenon that has been known for decades when performing cytogenetic analysis of chorionic villi [13–15].

Most laboratories adopt a minimum threshold of 4% cffDNA for yielding an accurate result by MPS [5]. Several factors influence the contribution of fetal cells to the total cell-free DNA. A low fetal DNA fraction has been attributed to early gestational age or high maternal weight [5, 16–18]. Also, prolonged (>24 hrs) storage of blood samples under suboptimal conditions prior to processing reduces the fetal DNA fraction because of an increase of maternal genomic DNA due to cell degradation [19, 20]. Furthermore, a reduction has been reported in the fetal DNA fraction in trisomy 13, trisomy 18, and monosomy X pregnancies as compared to euploid pregnancies [21, 22], but this has not been confirmed in all studies [23]. Finally, in cases of a mosaic placenta, the presence of euploid cells reduces the fetal DNA fraction contributed by the aneuploid cells. For example, if there is 50% mosaicism for a trisomy in the cytotrophoblast, a fetal DNA fraction of 10% is reduced to an effective fraction of 5% for detecting the trisomy [17]. There is little information on how frequently these parameters affect the clinical performance of NIPT. A review of 15 clinical validation studies, together incorporating more than 21,000 cases, shows that few of the trisomies are missed [6]. For trisomy 21, 3 out of 835 cases were false negative (0.36%), 6 out of 315 for trisomy 18 (1.90%) and 4 out of 60 for trisomy 13 (6.67%). In most of these studies, the fetal DNA fraction was not provided for the false negative cases, and follow-up by cytogenetic or molecular genetic investigation of the placenta and the newborn child was not included in the study design. Therefore, few data are available on the contribution of the diverse causes of false negative results. Problems with sample identity were reported in one study, leading to a false negative result in 2 out of 3,430 cases [24]. Published case reports of false negative NIPT results are summarized in Table 1, showing that, in cases with molecular or cytogenetic follow-up investigations, placental mosaicism was frequently involved and was the most likely cause of the false negative result. Here we report the cytogenetic follow-up of two novel false negative cases.

## 2. Case Reports

In neighboring countries of The Netherlands, commercial marketing of NIPT started in 2012, and several thousands of Dutch women, including women at low risk for fetal aneuploidy, have opted for “outsourced” NIPT testing via institutions abroad. The two cases described here came to our attention after a fetal anatomy scan at 20 weeks of gestation revealed anomalies indicative of trisomy 13 (Case 1) and trisomy 18 (Case 2). Case 1 has been referred to previously [35]. The relevant characteristics of these two cases are summarized in Table 2.

### 2.1. Case 1

In a 35-year-old, healthy woman (G1P0), first trimester combined testing for Down syndrome at 12 5/7 weeks of gestation showed a risk of 1/190 for trisomy 18; the nuchal translucency (NT) was 1.6 mm (1.18 MoM). She opted for NIPT to avoid potential complications of invasive testing. Blood was taken at 13 5/7 weeks of gestation and sent overseas via an intermediate party. Maternal weight and BMI (body mass index) were 59 kg and 22.0, respectively. NIPT results were available at 15 weeks and indicated that she was at “low risk” for each of the three common trisomies (<1/10,000). The cffDNA fraction was reported to be 8.8%. Ultrasound examination at 19 5/7 weeks showed a small male fetus with a right-sided cleft of the lip and alveolar ridge and cerebellar vermis hypoplasia. There were no signs of a vanishing or demised fetus nor of an empty, second sac. Two days later, the couple was counseled by a clinical geneticist. Amniocentesis was performed at 20 5/7 weeks. The patient consented to have a blood sample taken for NIPT prior to amniocentesis. The blood sample was processed and analyzed in the laboratory of our department, at the time performing a NIPT validation study. The result of quantitative fluorescence polymerase chain reaction (QF-PCR) based on DNA extracted from uncultured amniocytes was indicative of nonmosaic trisomy 13, and the couple was informed at 21 weeks. Karyotyping of G-banded metaphases of cultured amniocytes (12 clones,* in situ* method) showed a 47,XY,+13 karyotype in all metaphases. NIPT in our department using the SOLiD Wildfire was performed as described [36]; the result was consistent with trisomy 13 (*z*-score 25.5). The couple was counseled by the clinical geneticist for a second time and was supported by a bereavement counselor for decision making. The pregnancy was terminated at 22 weeks. Postpartum karyotyping of 32 metaphases of cultured fetal fibroblasts showed a 47,XY,+13 karyotype in all cells. The placenta was sampled at 9 representative approximately equidistant positions, representing 9 equally large sections. For each biopsy, mesenchymal and cytotrophoblast cells were separated as published earlier [37, 38] and 100 interphase nuclei of each cell type were investigated by fluorescence* in situ* hybridization (FISH). This showed high percentages of cells with trisomy 13 throughout the placenta in both cytotrophoblast (average 96%; range 91–100%) and mesenchyme (average 96%; range 92–100%). A similar result was seen for the umbilical cord (97%).

### 2.2. Case 2

A 40-year-old, healthy woman (G2P1) had blood taken for NIPT at 11 0/7 weeks of gestation. The blood sample was sent overseas via an intermediate party. The cffDNA fraction was reported to be 10.7% and the test result indicated that there was a “low risk” for each of the three common trisomies (<1/10,000). Maternal weight and BMI were 70 kg and 22.4, respectively. At ultrasound examination at 19 5/7 weeks, multiple anomalies were noted in a female fetus, including a strawberry skull, bilateral plexus cysts, a complex cardiac anomaly (large ventricular septal defect, Ebstein anomaly of the tricuspid valve, and abnormal pulmonary venous connection), and bilateral clenched fists and rocker bottom feet. There were no indications for presence of a vanishing twin nor of an empty sac. The next day amniocentesis was performed and the patient agreed to have a blood sample taken, prior to amniocentesis, for NIPT. Results of QF-PCR based on DNA from uncultured amniocytes were available at 20 weeks and were indicative of nonmosaic trisomy 18. The couple was counseled by a clinical geneticist and assisted by a bereavement counselor. Karyotyping of metaphases of 30 clones of cultured amniocytes showed a 47,XX,+18 karyotype in all clones. In addition, FISH showed trisomy 18 in all 52 metaphases investigated after trypsinization of cells cultured* in situ*. NIPT performed in our department using the SOLiD Wildfire [36] was also consistent with trisomy 18 (*z*-score 25.4). The pregnancy was terminated at 23 2/7 weeks after repeated counseling sessions. In cultured lymphocytes from fetal blood, trisomy 18 was detected in all 89 metaphases investigated. Ten biopsies were taken from the placenta, representing 10 equally sized sections, and investigated as described above. Eight biopsies had ≥70% trisomy 18 cells in both cytotrophoblast (average 78%; range 70–90%) and mesenchyme (average 78%; range 73–83%). In two biopsies the cytotrophoblast showed 64% and 69% trisomy 18, respectively, whereas mesenchyme was 88% and 80%, respectively. In the umbilical cord biopsy, we found 80% trisomy 18. Presence of 20–30% euploid cells in the cytotrophoblast would reduce the 10.7% fetal fraction to an effective fetal fraction of 7.5–8.5%.

## 3. Discussion

We describe two cases in which “outsourced” NIPT gave a “low risk” result (<1/10,000) in women carrying a trisomic fetus. The patients received the NIPT result between 12 and 15 weeks of gestation. When, at 20 weeks of gestation, multiple fetal anomalies were detected by ultrasound examination, amniocentesis was performed. Analysis by QF-PCR and karyotyping revealed trisomy 13 in one case and trisomy 18 in the other. These unexpected, discrepant results caused disbelief and distress to the families, requiring multiple counseling sessions.

Several companies in the USA and Europe are marketing MPS-based NIPT directly to the clinician (e.g., Ariosa Diagnostics, Natera, Verinata Health, the Sequenom Center for Molecular Medicine, and LifeCodexx). Validation studies in high risk pregnancies (elevated maternal age, increased risk from first trimester combined testing) showed high sensitivity and specificity for the detection of each of the three common trisomies [3, 4, 6–10]. False negative results are inherent to this technique that is based on quantification of sequence reads of cffDNA fragments originating from the cytotrophoblast. This can be explained in several ways. NIPT depends on a statistical assessment of the sequence reads. Therefore, cutoff values must be defined for discrimination between normal and abnormal results, and, as a consequence, NIPT is still considered a screening test and not a diagnostic test [11]. In addition, false negative results can be caused by a low fetal fraction, for example, when NIPT is done too early in gestation (<10 weeks) [5, 14, 17], in obese women [5, 16–18], in cases of suboptimal, prolonged storage of blood samples prior to processing [19, 20], or if there is an euploid vanishing twin that contributed to the cffDNA. Finally, large-scale evaluation of CVS showed that in 0.8–1% of cases there is confined placental mosaicism, with a different karyotype in cytotrophoblast cells, the source of cffDNA, than that in the fetus proper [13, 14]. In a retrospective study based on 52,673 pregnancies, placental mosaicism was predicted to be the likely cause of a false negative NIPT result in 1/136 trisomy 13, 1/64 trisomy 18, and 1/135 trisomy 21 cases [39].

To systematically explore the possible causes of the false negative results in our two cases we first looked at factors known to cause a low fetal DNA fraction. An early gestational age (<10 weeks) or elevated maternal weight was not implied and we have no indications for suboptimal storage or transport conditions of the blood samples. Second trimester ultrasound examination did not indicate the presence of a vanishing twin or an empty, second sac, and we assume that this was also the case at the time of blood sampling for NIPT. To look for mosaicism as an explanation, we examined multiple placental biopsies and karyotyped fetal cells. The results are summarized in Table 2. In Case 1, the proportion of nontrisomic cells in the cytotrophoblast was 0% or close to 0%. In Case 2, on average not more than 20–30% nontrisomic cells were present in the cytotrophoblast. Given a cell-free fetal DNA fraction of 10.7%, this percentage of nontrisomic cells would not have been large enough to lower the fraction of aneuploid, fetal DNA below 4%. Case 2 differs in this respect from the cases described by Canick et al. [17], Wang et al. [29], Gao et al. [25], and Mao et al. [26] in which much larger fractions of nontrisomic cells were found in placental biopsies (Table 1). Thus, there is no evidence that the false negative NIPT results in Cases 1 and 2 are due to placental mosaicism. Because the NIPT tests were carried out by a third party we could not verify sample identity (as required according to ISO 15189 [40]). We conclude that in both cases the root cause of the discrepant NIPT results could not be identified.

So, how should the problem of unexpected false negative NIPT results be handled in clinical practice? During pretest counseling it must be clearly explained to the patient that NIPT is based on DNA fragments from the placenta, not from the fetus, and that a false positive or false negative result may occur. This will enable pregnant women and their partners to make informed choices between NIPT and alternative options and allows to reinforce the usefulness of a fetal anatomy scan at 20 weeks of gestation. Furthermore, the presence of a demised cotwin should always be excluded since this might cause false negative (or false positive) results. In addition, a systematic investigation into the cause of discrepancies as described in this paper will be helpful not only to understand the limitations of NIPT but also to improve its performance in daily clinical practice. This investigation should include verification of sample identity as required according to ISO 15189 [40]. Finally, an international registry for systematic recording of all discordant NIPT results and their causes, as was done when CVS was introduced in prenatal diagnosis more than 30 years ago [13, 14], will provide insight into the frequency and causes of false negative and false positive NIPT results [11, 41]. In clinical practice, reported frequencies of false negative results show a surprisingly large and unexplained variation, ranging from 1/16,000 [28] or 1/9,000 [32] to 1/200 [33] consecutive cases from a mixed low and high risk population. In case of a trisomy detected by NIPT, CVS or amniocentesis can be offered for confirmation, depending on the gestational age. In case CVS is opted, both cytotrophoblast and mesenchymal cells should be investigated, and, even so, one must be aware of the possibility that a trisomy can be confined to the placenta [15, 39], a problem that does not play a role when analyzing amniotic fluid cells.

## Figures and Tables

**Table 1:** Survey of published cases of false negative NIPT by massive parallel shotgun sequencing with (molecular) cytogenetic follow-up included^1^.

Trisomy	Study [reference]	Indication for NIPT	Case, mat. age	Blood drawn at GA^2^	Fetal DNA fraction (effective fetal DNA fraction)	Result of NIPT	Karyotype	Explanation for false negative NIPT result
13	Canick et al. (2013) [17]	Ultrasound abnormalities	Case 1 34 yrs	14 w	6% (0.6%)	*z*-score 0.08 for chromosome 13	46,XX/47,XX,+13 in cultured amniocytes	Amniocentesis showed 10% mosaicism for a cell line with +13

18	Canick et al. (2013) [17]	Maternal age	Case 5 39 yrs	12 w	23% (10%)	*z*-score 0.22 for chromosome 18	47,XY,+21/48,XY,+18,+21 in CVS	CVS showed 45% mosaicism for a cell line with both +18 and +21

18	Gao et al. (2014) [25]	1/70 risk for tri-21 by combined 1st trim. test	43 yrs	13 w	7.4%	Low risk tri-13, tri-18, and tri-21, high risk XXX	48,XXX,+18 in cultured amniocytes	Estimated 20–30% of placental cells studied by FISH were +18, with QF-PCR showing variable levels of +18 cells across the placenta

18	Mao et al. (2014) [26]	1/313 risk for tri-18 by serum screening	22 yrs	17 w	11.6% (4.1%)	*z*-score 0.35 for tri-18; *z*-score 4.4 for tri-21	47,XX,+18 in cultured amniocytes	Placental biopsies showed on average 50% +21, 35% +18, and 15% normal cells, but at least one region had 61% +21 but only 22% +18

18	Pan et al. (2014) [27]	1/45 risk for tri-21 by combined test	24 yrs	18 w	Not provided	*t*-score −0.52 for tri-18; *t*-score −4.05 for X-chromosome	47,XX,+18 in cultured amniocytes, both by karyotyping and SNP-array	About 30% of placental cells studied by FISH were +18, and about 67% were 45, X; SNP-array was indicative of ~50% cells with +18

18	Zhang et al. (2015) [28]	1/360 risk for tri-21 by combined test	29 yrs	19 w	5.3% (1.1–2.2%)	Aneuploidy not detected	47,XY,+18 in products of conception	6 placental biopsies showed 20–40% cells with +18

18	Zhang et al. (2015) [28]	1/45 risk for tri-21 by combined test	24 yrs	20 w	9.5% (2.9%)	Indicative of 45,X	47,XX,+18 in cultured amniocytes	In placental tissue 30% of the cells showed +18 and 60% showed 45,X

21	Canick et al. (2013) [17]	Maternal age	Case 2 44 yrs	11 w	17% (1.7%)	*z*-score 2.03 for chromosome 21	46,XY[20]/47,XY,+21[2] in CVS	CVS showed 9% mosaicism for a cell line with +21

21	Canick et al. (2013) [17]	Maternal age	Case 3 41 yrs	12 w	9% (4.5%)	*z*-score −1.25 for chromosome 21	46,XY/47,XY,+21 in CVS	50% mosaicism by CVS; possibly this was lower in the placenta as a whole

21	Wang et al. (2013) [29]	1/370 risk for tri-21 by serum screening at 17 w	Case 1 32 yrs	18 w	15.6%	*z*-score 2.04^3^ for chromosome 21	46,XX,der(21;21)(q10;q10),+21 in fetal blood (cordocentesis)	Placental biopsies had 17%, 21%, 23%, and 53% cells with +21

21	Wang et al. (2013) [29]	2 spontaneous abortions	Case 2 35 yrs	18 w	19.7%	*z*-score 1.33 for chromosome 21	47,XY,+21 in cultured amniocytes	Placental biopsies had 2%, 51%, and 76% cells with +21

21	Smith et al. (2014) [30]	CAVCD^4^	32 yrs	20 w	Not provided	“Negative” for trisomy 21	47,XX,+21 in postnatal blood	No study of placenta or umbilical cord; no explanation provided

*Notes*. ^1^A false negative case of trisomy 21 was also mentioned by Wang et al. [31], 2 other false negative trisomy 21 cases were mentioned by Dar et al. [32], and another 6 false negative trisomy 21 cases were described by Zhang et al. [28]; a false negative case of trisomy 18 was mentioned by Beamon et al. [33], another one by Quezada et al. [34] and by Zhang et al. [28]; 3 false negative cases of tri-13 were reported by Quezada et al. [34]; all of these were without further investigation of the possible causes of the discrepant findings.

^2^GA: gestational age.

^3^In a repeat blood sample taken at 24 weeks the *z*-score for chromosome 21 was 4.0 and trisomy-21 was reported as a result.

^4^CAVCD: complete atrioventricular canal defect.

**Table 2:** Demographics and summary of laboratory testing in two cases with false negative NIPT.

Case; age (status)	BMI	Medication, alcohol, and drugs	1st trimester combined screening (NT)	Blood for NIPT drawn at GA^1^	Result of NIPT issued by a third party				Pregnancy outcome; cytogenetic follow-up
					Fetal fraction	Tri-13	Tri-18	Tri-21	
Case 1 35 yrs (G1P0)	22.0	Negative	1 : 190 risk for trisomy-18 (NT 1.6 mm)	13 5/7	8.8%	<1/10,000	<1/10,000	<1/10,000	Amniocentesis at 21 weeks showed a nonmosaic 47, XY, +13 karyotype; sampling of placenta (9 biopsies) gave no evidence for the presence of euploid cells

Case 2 40 yrs (G2P1)	22.4	Negative	Not performed	11 0/7	10.7%	<1/10,000	<1/10,000	<1/10,000	Amniocentesis at 20 weeks showed a nonmosaic 47, XX, +18 karyotype; sampling of placenta (10 biopsies) showed that a maximum of 20–30% euploid cells may have been present in the cytotrophoblast

*Note*. ^1^GA: gestational age.
